# Expanded newborn screening for inborn errors of metabolism in Isfahan Province: the first two-year report from Central Iran

**DOI:** 10.1186/s13023-026-04539-4

**Published:** 2026-08-08

**Authors:** Amir Masoud Mohajeri, Mehrshad Safaeian, Mohammad Shahpiri, Mohsen Akbari Dehkordi, Mehrdad Zeinalian

**Affiliations:** 1 Student Research Committee, School of Medicine, Isfahan University of Medical Sciences, Isfahan, Iran; 2 Department of Genetics and Molecular Biology, Isfahan University of Medical Sciences, Isfahan, Iran; 3 Department of Noncommunicable Diseases, Deputy of Health, Isfahan University of Medical Sciences, Isfahan, Iran; 4 Department of Genetics and Molecular Biology, School of Medicine, Isfahan University of Medical Sciences, Isfahan, Iran

**Keywords:** Metabolism, Inborn errors, Neonatal screening, Isfahan, Iran

## Abstract

**Background:**

Inborn errors of metabolism cause significant infant morbidity and mortality. Birth prevalence is elevated in the Eastern Mediterranean region, influenced by consanguinity. This study presents the first findings of an expanded newborn screening program for IEMs in Isfahan Province, Central Iran. In this cross-sectional study (2023–2025), 71,004 neonates were screened via tandem mass spectrometry (MS/MS) of dried blood spots for 50 metabolic disorders. Positive cases underwent confirmatory testing. Data on demographics and consanguinity were analyzed.

**Results:**

From 71,004 newborns, 48 cases were confirmed, corresponding to a prevalence of 67.6 per 100,000 live births, including organic acid, amino acid, and fatty acid oxidation disorders. The most frequent disorders included Short-chain acyl-CoA dehydrogenase (SCAD) deficiency, benign hyperphenylalaninemia (HPA), and 2-Methylbutyryl-CoA dehydrogenase (SBCAD) and 3-Methylcrotonyl-CoA carboxylase (3MCC) deficiencies. Consanguinity was a major risk factor, present in 56.3% of cases (Relative Risk = 2.05). All mortality (10.4%) occurred in the organic acid disorder group. Birth prevalence was highest in smaller towns (e.g., Khansar: 384.6/100,000).

**Conclusion:**

The high prevalence of IEMs in Isfahan, linked to consanguinity and a distinct disorder profile, underscores the critical importance of expanded NBS. The findings advocate for integrated public health strategies, including genetic counseling and targeted screening, to reduce the disease burden in this region.

## Background

Inborn errors of metabolism (IEMs) represent a heterogeneous group of more than 500 genetic conditions that often result in the disruption of normal biochemical pathways. They can lead to organ dysfunction and diverse clinical manifestations from the neonatal period [1, 2]. Although each individual disorder is rare, their combined prevalence is relatively high [2]. These monogenic disorders typically result from reduced activity or complete absence of a specific enzyme involved in an intermediate metabolic pathway. This enzymatic deficiency may lead to the accumulation of toxic metabolites or a shortage of essential biochemical products, causing a wide spectrum of clinical manifestations, from asymptomatic cases to severe, progressive, and potentially fatal diseases [3–5].

Early recognition is therefore critical, as delayed diagnosis and treatment can result in irreversible outcomes such as neurological impairment, intellectual disability, and death [3, 4]. Accurate diagnosis commonly requires a high index of clinical suspicion together with access to advanced laboratory technologies and confirmatory testing — resources that are often limited in low- and middle-income countries [4, 6].

Recent population-based studies highlight the significant global impact and regional variation of IEMs. A systematic review covering nearly four decades (1980–2017) estimated the global IEM prevalence to be roughly 50.9 cases per 100,000 live births. The highest rates were reported in the Eastern Mediterranean region, where a high prevalence of consanguineous marriages may partly explain this elevation [1]. A seven-year study in China identified phenylketonuria (PKU), methylmalonic acidemia (MMA), and short-chain acyl-CoA dehydrogenase (SCAD) deficiency as the most frequent diagnoses [7]. A three-year screening program in Fars, Iran, yielded a high prevalence of 100 per 100,000 live births for IEMs, with aminoacidopathies being the most frequent type [8]. Furthermore, the reported prevalence of PKU is about 16.6 per 100,000 live births in Iran [9]. Together, these studies highlight the need for expanded newborn screening strategies, particularly in populations with genetic and demographic factors that increase the risk of autosomal-recessive disorders.

Newborn screening (NBS) has long been recognized as a highly effective public health strategy, allowing the detection of inborn metabolic disorders before the onset of clinical symptoms [10]. Through early medical or nutritional management, NBS offers the possibility to modify disease progression and prevent serious or life-threatening outcomes. Consequently, the advancement of screening technologies has become essential for improving the effectiveness of NBS. The introduction and widespread use of tandem mass spectrometry (MS/MS) have revolutionized NBS programs worldwide, providing a highly sensitive and specific tool capable of identifying multiple metabolic conditions rapidly in a single analytical run [7, 11].

Accordingly, expanded newborn screening has become a rational and cost-effective public health approach in many populations [12–14]. In Iran—with its remarkable geographic diversity, distinct genetic background, and rich ethnic and cultural heterogeneity—a national newborn screening program for hyperphenylalaninemia has been established for more than a decade [9]. In recent years, several provinces have expanded screening programs through the introduction of MS/MS to detect a broader range of metabolic disorders [8, 15, 16]. In 2021, a pilot program for screening 50 IEMs was initiated in just five counties of Isfahan province, but since September 2023, the program has been implemented across all counties affiliated with Isfahan University of Medical Sciences. This is while the program continues to expand across the country, including in Isfahan Province. This expansion represents an important step toward improving early detection in populations with similar genetic and demographic characteristics.

## Materials and methods

### Study design

This cross-sectional descriptive study was conducted in Isfahan Province, Iran, from September 2023 to May 2025. All neonates born in hospitals across counties affiliated with Isfahan University of Medical Sciences who underwent neonatal screening for inborn errors of metabolism (IEMs) were enrolled. The counties included: Isfahan, Ardestan, Borkhar, Buin Miandasht, Tiran and Karvan, Chadegan, Khomeyni Shahr, Khansar, Khur and Biabanak, Dehaqan, Semirom, Shahin Shahr and Meymeh, Shahreza, Fereydan, Fereydunshahr, Falavarjan, Mobarakeh, Golpayegan, Najafabad, Varzaneh, Lenjan, Natanz, Kuhpayeh, Nain, Harand, and Jarghouyeh. Newborns with abnormal metabolic screening results (defined as biochemical markers exceeding established cutoff values indicative of potential IEMs) were included if they received a confirmed diagnosis through follow-up evaluations. Infants without complete documentation or confirmatory results (including those who died before completing the experimentations) were excluded.

### Newborn screening & sample collection

In this program, all neonates underwent screening for 50 inherited metabolic disorders within 3–5 days of birth. The targeted disorders in this screening program are categorized into three groups: Amino Acid disorders, Fatty Acid disorders, and Organic Acid disorders. Each of these groups and their specific disorders are detailed in (Table 1). These disorders were selected based on their clinical significance, prevalence, treatability with available therapies, and the feasibility of diagnosis through cost-effective laboratory methods. Initially, a physician, midwife, nurse, or a trained specialist at the hospital completed the newborn’s information, including: full name, sex, date and time of birth, date and time of sample collection, mother’s full name, hospital name and location, type of parental relationship (consanguineous or non-consanguineous), and parental contact number. Subsequently, the responsible health worker collected a few drops of blood from the newborn’s heel onto a filter paper, forming a dried blood spot (DBS). The sample was then sent to the reference laboratory in Isfahan for analysis.

**Table 1:** Targeted inborn errors of metabolism included in newborn screening in Isfahan Province, Iran

Amino Acid Disorders	Fatty Acid Oxidation Disorders	Organic Acid Disorders
Argininosuccinic Aciduria	Primary Carnitine Deficiency / Carnitine Transporter Defect	Propionic Acidemia
Citrullinemia Type 1	Medium Chain Acyl-CoA Dehydrogenase Deficiency	Methylmalonic Acidemia: methylmalonyl-CoA mutase
Maple Syrup Urine Disease	Very Long Chain Acyl-CoA Dehydrogenase Deficiency	Methylmalonic Acidemia: Cobalamin Disorders
Homocystinuria	Long Chain 3-Hydroxyacyl-CoA Dehydrogenase Deficiency	Isovaleric Acidemia
Phenylketonuria Classic	Trifunctional Protein Deficiency	3-Methylcrotonyl-CoA Carboxylase Deficiency
Tyrosinemia Type 1	Short Chain Acyl-CoA Dehydrogenase Deficiency	3-Hydroxy-3-Methylglutaric Aciduria
Argininemia	Medium/Short Chain Acyl-CoA Dehydrogenase Deficiency	Holocarboxylase Synthase Deficiency
Citrullinemia Type 2	Glutaric Acidemia Type 2	β-Ketothiolase Deficiency
Hypermethioninemia	Medium Chain ketoacyl-CoA Thiolase Deficiency	Glutaric Acidemia Type 1
Benign Hyperphenylalaninemia	2,4-Dienoyl-CoA Reductase Deficiency	Methylmalonic Acidemia, cblC form
Biopterin Biosynthesis Defect (2 conditions)	Carnitine Palmitoyltransferase 1 Deficiency	Methylmalonic Acidemia, cblD form
Biopterin Regeneration Defect (2 conditions)	Carnitine Palmitoyltransferase 2 Deficiency	Malonic Acidemia
Non-Ketotic Hyperglycinemia	Carnitine Acylcarnitine Translocase Deficiency	Isobutyrylglycinuria
Ornithine Transcarbamylase Deficiency		2-Methylbutyrylglycinuria
Carbamoyl Phosphate Synthetase 1 Deficiency		3-Methylglutaconic Aciduria
Tyrosinemia Types 2,3 (2 conditions)		2-Methyl-3-hydroxybutyric Aciduria
Glycine N-methyltransferase (GNMT) deficiency		Ethylmalonic Encephalopathy
Adenosylhomocysteine hydrolase deficiency		
HHH Syndrome		
Pyruvate carboxylase deficiency		

### Mass spectrometry & metabolite analysis

Laboratory analysis for IEMs was conducted using advanced mass spectrometric techniques on dried blood spot (DBS) samples. The primary analytical method employed was tandem mass spectrometry (LC-MS/MS or HPLC–MS/MS). This approach enables the simultaneous quantification of multiple metabolites across three primary biochemical categories: amino acids, acylcarnitines, and organic acids. It offers high analytical sensitivity and specificity, a broad dynamic range, and rapid analysis, collectively facilitating the detection of a wide spectrum of metabolic disorders.

The amino acid panel included S-Adenosylhomocysteine, Arginine, Argininosuccinic acid, Phenylalanine, Biopterin metabolites, Glutamine, Citrulline, Glycine, Methionine, Homocysteine, Ornithine, Leucine, Isoleucine, Valine, Alanine, Lactate, Pyruvate, Tyrosine, and Succinylacetone. Fatty acid oxidation disorders were assessed through the measurement of acylcarnitine and carnitine species such as C0 (free carnitine), C6, C6DC, C4, C4OH, C4DC, C8, C8:1, C10, C10:1, C14, C14:1, C14:2, C14OH, C16, C16:1, C16OH, C16:1OH, C18, C18:1, C18:2, C18OH, C18:1OH, propionylcarnitine, and ketoacids. The organic acid panel encompassed 2-Methyl-3-hydroxybutyric acid, 2-Methylbutyryl glycine, 3-Hydroxy-3-Methylglutaric acid, C5-OH, 3-Methylglutaconic acid, C5:1, Ethylmalonic acid, Glutaric acid, C5DC, biotin-dependent metabolites, Isobutyrylglycine, Isovalerylglycine, Malonic acid, C3, Methylmalonic acid, and Propionylcarnitine. All metabolites were quantified using validated mass spectrometry-based protocols, ensuring robust sensitivity and specificity for accurate neonatal detection of IEMs.

### Risk stratification & screening workflow

The neonatal screening workflow for IEMs employs a tiered, risk-based algorithm. Prior to program implementation, the laboratory team established normal ranges, gray zones, and pathological cutoffs based on guidelines and pilot studies conducted on a healthy neonatal population. Infants are categorized into three groups: Healthy (low risk), Suspicious (medium risk), and High risk, based on the outcomes of screening assessments.

Newborns with initial screening results falling within the established normal reference range were classified as low-risk and continued routine follow-up as healthy infants, indicating that the newborn was not affected by any of the disorders included in the screening panel. Newborns with initial results in the pathologic interval (high-risk) were immediately referred to Amin Hospital in Isfahan (a tertiary center) for comprehensive assessment by pediatric endocrinology and metabolic specialists. Conversely, those with initial results within the suspicious or borderline interval (medium-risk) were recalled for a second DBS specimen to be reevaluated using MS/MS to confirm the initial findings. If the second test returned values within normal limits, infants were discharged to routine care. However, persistently abnormal or borderline repeat results prompted referral for specialized evaluation like high-risks. This structured approach ensured early identification of true positive cases while minimizing unnecessary referrals and parental anxiety.

### Diagnostic & confirmatory testing

At this stage, clinicians conduct a comprehensive medical and family history, perform a thorough physical examination, and request confirmatory laboratory tests. These tests are selected by the assigned expert according to ACMG guidelines and performed at reference Confirmatory laboratory in Isfahan or Shiraz (Based on the condition, type of disease and required diagnostic tests). The tests may include blood gas evaluation, serum ammonia measurement, liver function tests, routine biochemistry panel, and urinary organic acid analysis via GC/MS. Targeted enzymatic assays may also be performed when clinically indicated. Additionally, echocardiography, electrocardiography, magnetic resonance imaging, and standardized neurodevelopmental assessments may be performed to support diagnostic confirmation and characterize disease severity.

Genetic confirmation is pursued when biochemical or clinical findings remain suggestive. Typically, next-generation sequencing (NGS) panels targeting IEM-associated genes are employed, followed by Sanger sequencing to validate pathogenic variants. In certain settings, access to molecular testing may be limited by the family’s financial resources. By integrating biochemical and genomic data, a definitive classification can be achieved. Once pathogenic variants or disease-specific metabolic and clinical signatures are confirmed, the diagnosis is established.

### Early intervention & follow-Up

**Fig. 1:**
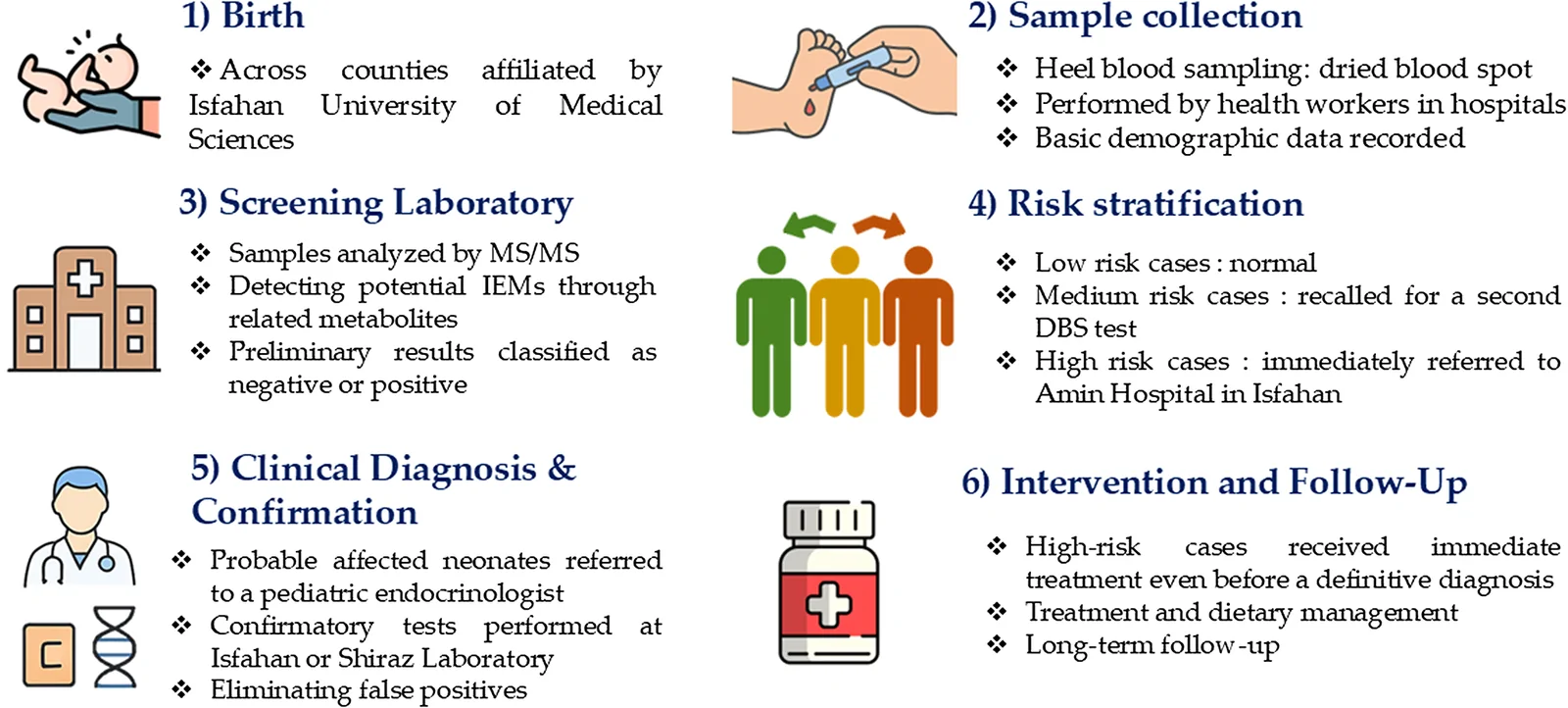
Workflow of the newborn screening program for IEMs in Isfahan Province, Iran, Data Management and Study Population showing steps from birth and sample collection to intervention and follow-up

High-risk infants received immediate treatment prior to definitive diagnosis to prevent metabolic decompensation. Depending on the suspected condition, interventions may include dietary protein restriction, disease-specific medical foods, cofactor therapy, or ammonia-lowering agents. Following their diagnosis, infants are enrolled in a structured long-term follow-up program. This program encompasses regular metabolic assessments, neurodevelopmental monitoring, and comprehensive clinical supervision. Throughout this process, parental engagement is maintained to ensure treatment adherence, biochemical stability, and ongoing counseling. Ultimately, this approach results in enhanced long-term outcomes for affected newborns. (Fig. 1)

In this process, all relevant information was systematically recorded using standardized forms. These forms captured demographic characteristics such as sex, gestational age, birth weight, city of residence, and parental consanguinity. Additionally, clinical findings, biochemical and molecular test results, and the final confirmed diagnosis were documented.

### Statistical analysis

Birth prevalence was determined by dividing the number of diagnosed cases by the total number of live births during the study period and multiplying the result by 100,000. The association between parental consanguinity and IEMs was assessed using relative risk (RR). Families were classified as consanguineous or non-consanguineous. RR and its 95% confidence interval (CI) were calculated using the standard error of the natural logarithm of RR.

$$\:SE[\mathrm{l}\mathrm{n}(RR\left)\right]=\sqrt{\frac{1}{a}-\frac{1}{a+b}+\frac{1}{c}-\frac{1}{c+d}}$$

Where a and b represent the number of affected and unaffected individuals in consanguineous families, and c and d correspond to affected and unaffected individuals in non-consanguineous families. Statistical significance was assessed using a two-sided Z-test, and a p-value < 0.05 was considered indicative of a statistically significant association. Additionally, to estimate the uncertainty in prevalence, 95% confidence intervals (CIs) were calculated using the Exact Poisson method, which is the recommended approach when dealing with low case counts or rare disorders. Analyses were conducted using SPSS version 24 (IBM Corp., Armonk, NY). Categorical variables were presented as frequencies and percentages.

### Ethical considerations

This study protocol was reviewed and approved by the Ethics Committee of Isfahan University of Medical Sciences (Approval code: IR.MUI.MED.REC.1404.124.).

## Results

### Prevalence patterns and distribution of screening-detected inborn errors of metabolism

**Fig. 2:**
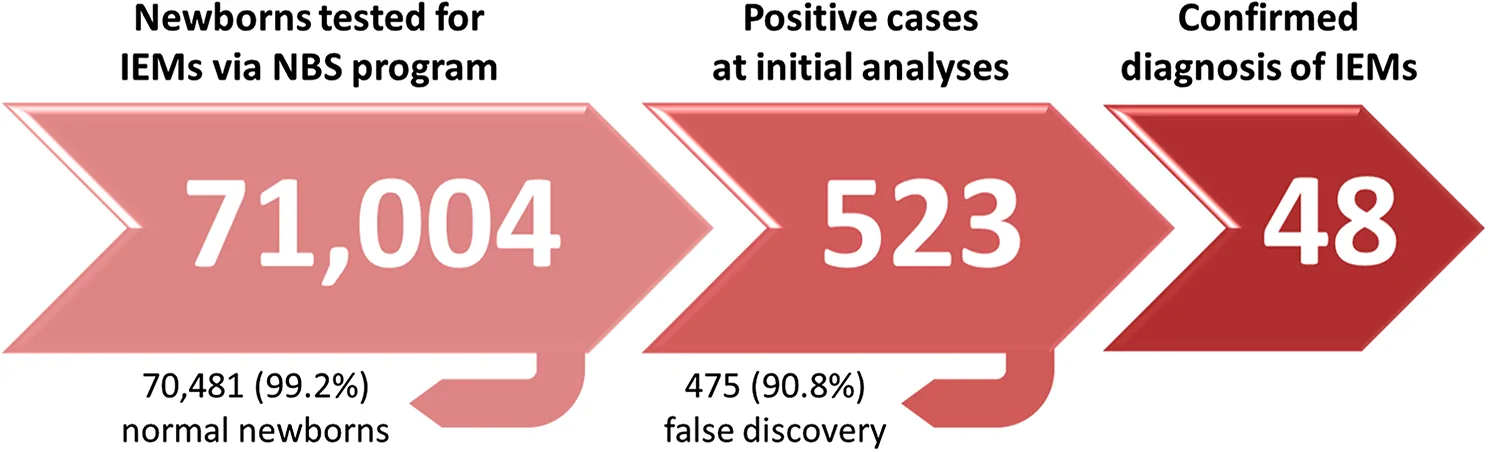
Flowchart of the expanded newborn screening program in Isfahan Province. A total of 71,004 newborns underwent screening, among whom 523 showed positive results at initial analyses, and 48 cases were confirmed with IEMs after diagnostic testing

During the study period, 71,004 newborns underwent screening. Out of these, 523 suspected cases were identified through initial analysis. Subsequently, 48 patients were confirmed to have inborn metabolic disorders, yielding a positive predictive value of 9.2% and a false discovery rate of 90.8%, as detailed in (Fig. 2).

**Fig. 3:**
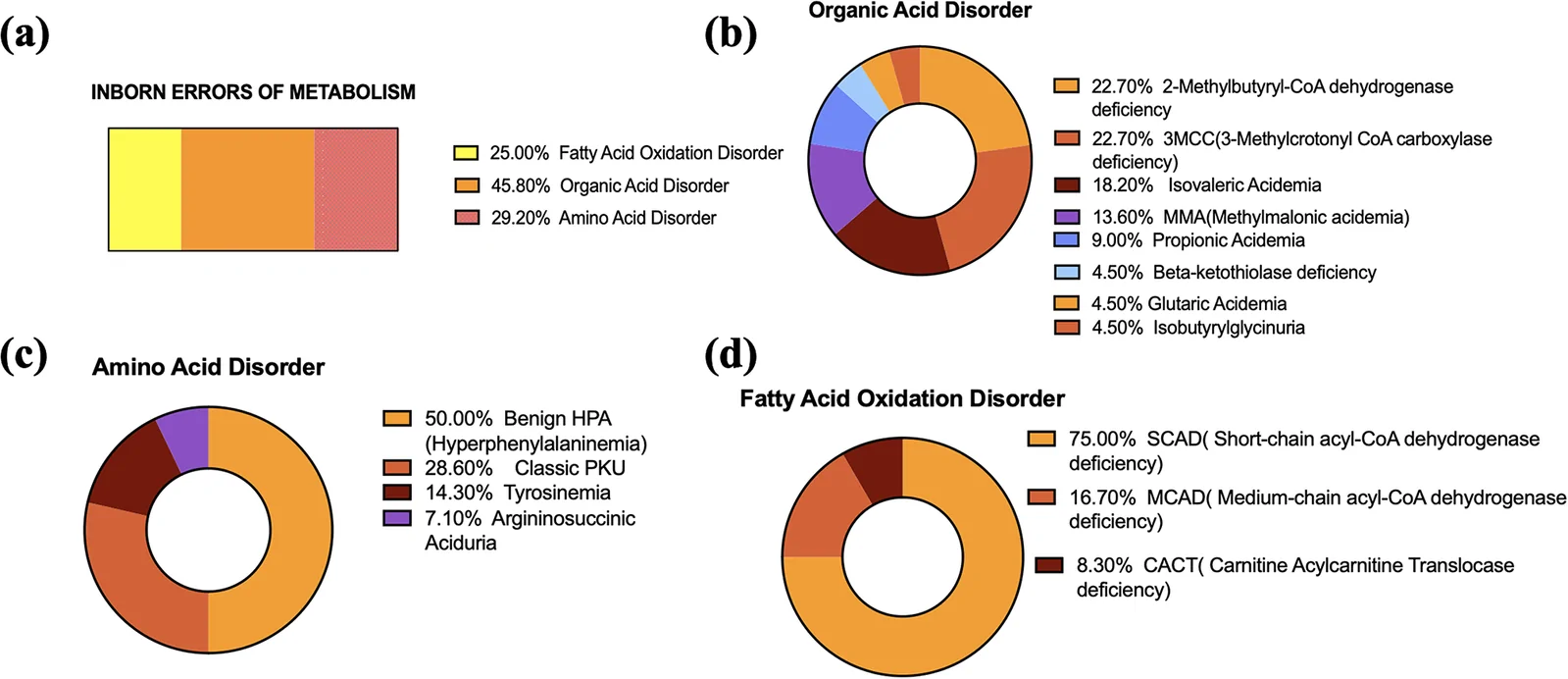
Distribution patterns of inborn errors of metabolism (IEMs) in Isfahan in 2023–2025. (**a**) overall proportions of the three main categories; (**b**) composition of organic acid disorders (OAD); (**c**) composition of amino acid disorders (AAD); and (**d**) composition of fatty acid oxidation disorders (FAOD)

48 cases of Inborn Errors of Metabolism (IEMs) were detected, resulting in an overall prevalence of 67.6 per 100,000 live births. Patients were categorized into three primary groups: Organic Acid Disorders (OAD, *n* = 22, 45.8%; birth prevalence 31 per 100,000), Amino Acid Disorders (AAD, *n* = 14, 29.2%; birth prevalence 19.7 per 100,000), and Fatty Acid Oxidation Disorders (FAOD, *n* = 12, 25%; birth prevalence 16.9 per 100,000) (Table 2). Within the OAD group, the most prevalent disorders were 2-Methylbutyryl-CoA dehydrogenase deficiency and 3-Methylcrotonyl-CoA carboxylase (3MCC) deficiency (each *n* = 5, 22.7%), followed by Isovaleric Acidemia (*n* = 4, 18.2%). Rare disorders included Beta-Ketothiolase deficiency, Glutaric Acidemia, and Isobutyrylglycinuria (each 4.5%). The male-to-female ratio was 1.44, twin births occurred in 3 cases (13.6%), most patients were Iranian (90.9%), and 5 patients (22.7%) died. In the AAD group, benign hyperphenylalaninemia (HPA, *n* = 7, 50%) and classic PKU (*n* = 4, 28.6%) were the most common disorders. Sex distribution was equal, most patients were Iranian (92.9%), and no deaths were reported. Among the FAOD group, SCAD deficiency (*n* = 9, 75%) was the most prevalent, followed by MCAD (*n* = 2, 16.7%) and CACT deficiency (*n* = 1, 8.3%). Sex distribution was equal, twin births occurred in 2 cases (16.7%), most patients were Iranian (75%), and no mortality was observed. Overall, there was a slight male predominance (54.2%), twin births were rare (10.4%), and mortality was confined to the OAD group (10.4% of all patients). (Fig. 3); (Table 2).

**Table 2:** Clinical and epidemiological characteristics of patients with inborn errors of metabolism and their subtypes

Inborn Errors of Metabolism	Cases (*n*)	Prevalence	Sex		Birth Weight (kg) (mean)	Maternal Age (year) (mean)	Twins (*n*)	Nationality		Consanguinity			Death (*n*)
			Male	Female				Iranian	Non-Iranian	First cousin	Second cousin	None-familial	
Organic Acid Disorder	22 (45.8%)	31 in 100,000 (95% CI: 19.42–46.91)	13 (59.1%)	9 (40.9%)	2.7	30.9	3 (13.6%)	20 (90.9%)	2 (9.1%)	7 (31.8%)	5 (22.7%)	9 (40.9%)	5 (22.7%)
2-Methylbutyryl-CoA dehydrogenase deficiency	5 (22.7%)	7 in 100,000 (95% CI: 2.29–16.43)	3 (60%)	2 (40%)	2.68	28	0 (0%)	4 (80%)	1 (20%)	1 (20%)	2 (40%)	2 (40%)	0 (0%)
3MCC (3- Methylcrotonyl CoA carboxylase deficiency)	5 (22.7%)	7 in 100,000 (95% CI: 2.29–16.43(	3 (60%)	2 (40%)	3.06	34.75	0 (0%)	4 (80%)	1 (20%)	1 (20%)	0 (0%)	4 (80%)	2 (40%)
Isovaleric Acidemia	4 (18.2)	5.6 in 100,000 (95% CI: 1.53–14.42)	3 (75%)	1 (25%)	1.9	29.25	3 (75%)	4 (100%)	0 (0%)	1 (25%)	1 (25%)	2 (50%)	1 (25%)
MMA (Methylmalonic acidemia)	3 (13.6%)	4.2 in 100,000 (95% CI: 0.87–12.35)	2 (66.7%)	1 (33.3%)	2.8	29	0 (0%)	3 (100%)	0 (0%)	0 (0%)	2 (66.7%)	1 (33.3%)	1 (33.3%)
Propionic Acidemia	2 (9%)	2.8 in 100,000 (95% CI: 0.34–10.18)	1 (50%)	1 (50%)	3.15	33	0 (0%)	2 (100%)	0 (0%)	2 (100%)	0 (0%)	0 (0%)	1 (50%)
Beta-ketothiolase deficiency	1 (4.5%)	1.4 in 100,000 (95% CI: 0.04–7.8)	1 (100%)	0 (0%)	3	42	0 (0%)	1 (100%)	0 (0%)	1 (100%)	0 (0%)	0 (0%)	0 (0%)
Glutaric Acidemia	1 (4.5%)	1.4 in 100,000 (95% CI: 0.04–7.8)	0 (0%)	1 (100%)	2.9	26	0 (0%)	1 (100%)	0 (0%)	1 (100%)	0 (0%)	0 (0%)	0 (0%)
Isobutyrylglycinuria	1 (4.5%)	1.4 in 100,000 (95% CI: 0.04–7.8)	0 (0%)	1 (100%)	NA	NA	0 (0%)	1 (100%)	0 (0%)	NA	NA	NA	0 (0%)
Amino Acid Disorder	**14** **(29.2%)**	**19.7 in 100,000** **(95% CI: 10.78–33.08)**	**7** **(50%)**	**7** **(50%)**	**3.1**	**31.54**	**0** **(0%)**	**13** **(92.9%)**	**1** **(7.1%)**	**4** **(28.6%)**	**3** **(21.4%)**	**7** **(50%)**	**0** **(0%)**
Benign HPA (Hyperphenylalaninemia)	7 (50%)	9.9 in 100,000 (95% CI: 3.96–20.31)	4 (57.1%)	3 (42.9%)	3.16	31.5	0 (0%)	7 (100%)	0 (0%)	0 (0%)	2 (28.6%)	5 (71.4%)	0 (0%)
Classic PKU (Phenylketonuria)	4 (28.6%)	5.6 in 100,000 (95% CI: 1.53–14.42)	2 (50%)	2 (50%)	3.1	29.67	0 (0%)	4 (100%)	0 (0%)	3 (75%)	0 (0%)	1 (25%)	0 (0%)
Tyrosinemia	2 (14.3%)	2.8 in 100,000 (95% CI: 0.34–10.18)	0 (0%)	2 (100%)	3.35	35	0 (0%)	1 (50%)	1 (50%)	0 (0%)	1 (50%)	1 (50%)	0 (0%)
Argininosuccinic Aciduria	1 (7.1%)	1.4 in 100,000 (95% CI: 0.04–7.85)	1 (100%)	0 (0%)	2.3	34	0 (0%)	1 (100%)	0 (0%)	1 (100%)	0 (0%)	0 (0%)	0 (0%)
Fatty Acid Oxidation Disorder	**12** **(25%)**	**16.9 in 100,000** **(95% CI**: **8.73–29.52)**	**6** **(50%)**	**6** **(50%)**	**3**	**31.3**	**2** **(16.7%)**	**9** **(75%)**	**3** **(25%)**	**8** **(66.7%)**	**0** **(0%)**	**4** **(33.3%)**	**0** **(0%)**
SCAD (Short-chain acyl-CoA dehydrogenase deficiency)	9 (75%)	12.7 in 100,000 (95% CI: 5.80–24.06)	5 (55.6%)	4 (44.4%)	3	32.14	2 (22.2%)	7 (77.8%)	2 (22.2%)	6 (66.7%)	0 (0%)	3 (33.3%)	0 (0%)
MCAD (Medium-chain acyl-CoA dehydrogenase deficiency)	2 (16.7%)	2.8 in 100,000 (95% CI: 0.34–10.18)	1 (50%)	1 (50%)	3.15	27.5	0 (0%)	1 (50%)	1 (50%)	1 (50%)	0 (0%)	1 (50%)	0 (0%)
CACT (Carnitine Acylcarnitine Translocase deficiency)	1 (8.3%)	1.4 in 100,000 (95% CI: 0.04–7.85)	0 (0%)	1 (100%)	3	33	0 (0%)	1 (100%)	0 (0%)	1 (100%)	0 (0%)	0 (0%)	0 (0%)
Total	**48** **(100%)**	**67.6 in 100,000** **(95% CI: 50.30–89.40)**	**26** **(54.2%)**	**22** **(45.8%)**	**2.91**	**31.18**	**5** **(10.4%)**	**42** **(87.5%)**	**6** **(12.5%)**	**19** **(39.6%)**	**8** **(16.7%)**	**20** **(41.7%)**	**5** **(10.4%)**

### Maternal age distribution among patients

**Fig. 4:**
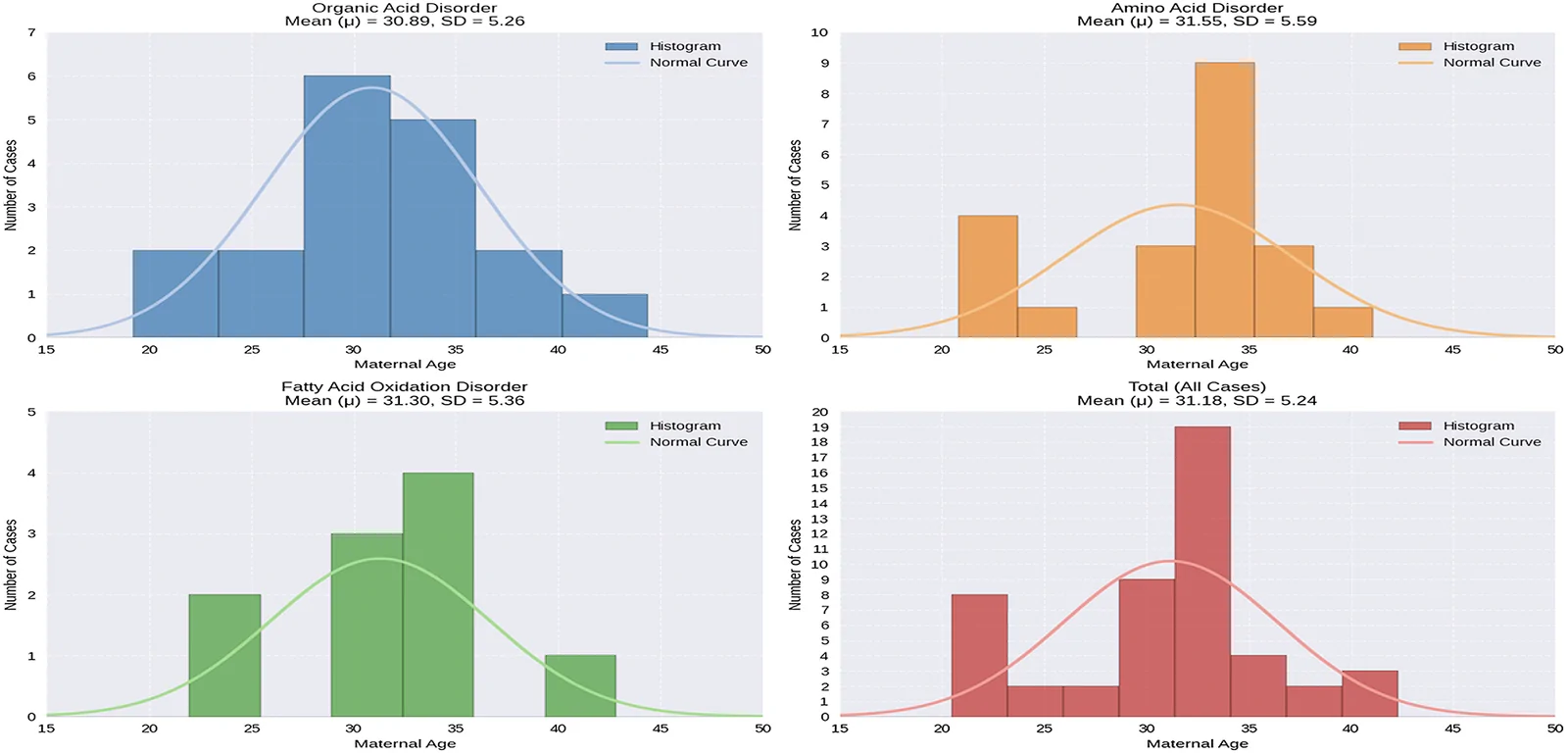
Maternal age distribution among patients. This figure illustrates the frequency of IEM cases categorized by maternal age groups for each major metabolic disorder, as well as the overall cohort. A line represents the fitted normal distribution for each group, providing a visual approximation of maternal age distribution patterns

The distribution of maternal age among patients with Inborn Errors of Metabolism (IEMs) reveals that no cases were reported in mothers younger than 18 years. The majority of IEM cases occurred in mothers aged 30–34 years (39.6%), followed by 18–24 years (14.6%) and mothers with missing age data (NA, 18.8%). Among Organic Acid Disorders (OADs), the highest frequency was observed in the 30–34 year age group (36.4%). Amino Acid Disorders (AADs) were most common in mothers aged 30–34 (28.6%) and 35–39 years (28.6%). Fatty Acid Oxidation Disorders (FAODs) predominated in the 30–34 year age range (58.3%). Overall, these findings indicate that maternal age of 30–34 years with the highest occurrence of IEMs. In contrast, mothers who are very young (< 18 years) or older (> 45 years) contributed minimally, consistent with the typical reproductive age distribution in the region and showing no apparent age-related disease pattern. (Fig. 4)

### Birth weight distribution among patients

**Fig. 5:**
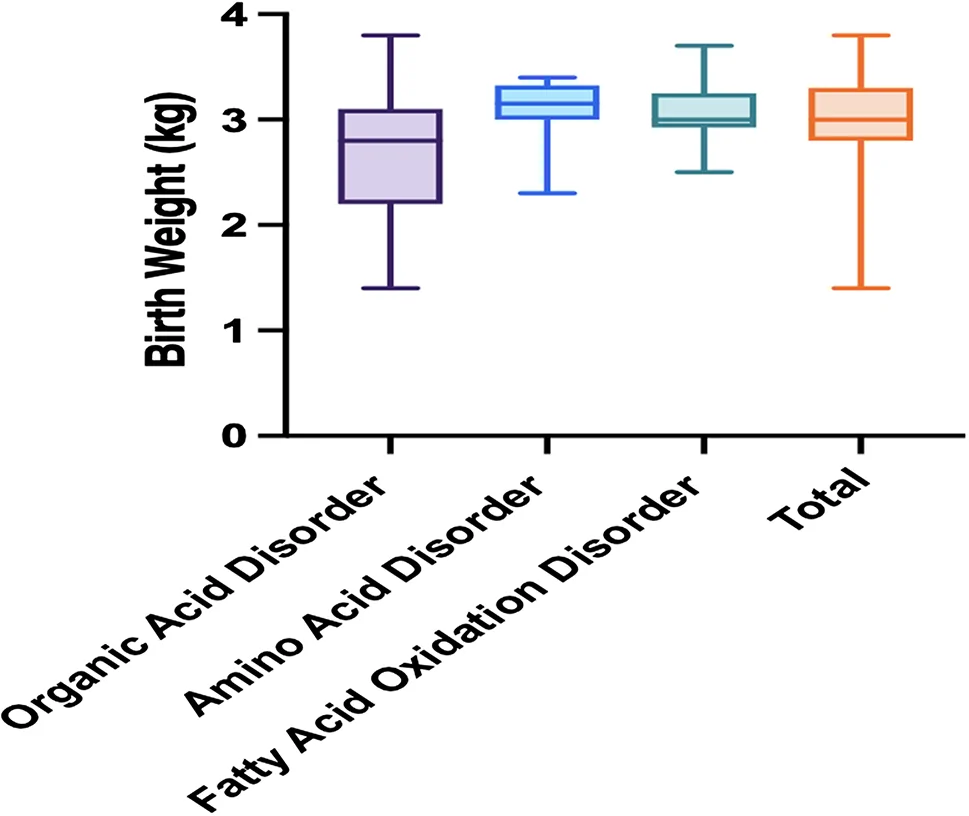
Birth weight distribution among neonates with Inborn Errors of Metabolism (IEMs). Boxplots illustrate the variation in birth weight across the three major categories of metabolic disorders. Median birth weights were approximately 2.7 kg for Organic Acid Disorders, 3.1 kg for Amino Acid Disorders, and 3.0 kg for Fatty Acid Oxidation Disorders. The Amino Acid Disorder group exhibited the narrowest variability, whereas Organic Acid Disorders showed greater dispersion. Overall, birth weights ranged from 1.4 to 3.8 kg, with most infants falling within the normal birth-weight range (2.5–4.0 kg). These patterns indicate potential subgroup-specific influences on fetal growth, particularly in Organic Acid Disorders

The birth weight o‌f 48 patients with Inborn Errors of Metabolism was assessed. These data illustrate that the majority of infants were born with normal birth weight (NBW, 2500–4000 g), accounting for 83.3% of cases. Low birth weight (LBW, 1500–2499 g) comprised 12.5% of the cohort, while very low birth weight (VLBW, < 1500 g) was observed in a single case (2.1%). No cases of macrosomia (> 4000 g) were reported, and birth weight data were missing for one patient (2.1%). Analysis by metabolic subgroup revealed distinct patterns. In Organic Acid Disorders (OADs), 68.2% of infants had NBW, 22.8% had LBW, and 4.5% had VLBW, with one case missing data. (Fig. 5)

Amino Acid Disorders (AADs) were predominantly associated with NBW (92.9%), with only one LBW case and no VLBW or macrosomic cases, indicating minimal impact on fetal growth. In contrast, all infants with Fatty Acid Oxidation Disorders (FAODs) were born with NBW, consistent with normal fetal growth patterns. In contrast, OADs showed a relatively higher proportion of LBW and VLBW infants. Notably, several of the LBW cases in this group occurred among infants with Isovaleric Acidemia, which may indicate a possible growth-related vulnerability in this specific disorder. These observations may support closer prenatal follow-up for pregnancies at risk of OADs.

### Consanguineous marriages distribution among patients

Consanguineous marriages were prevalent among patients with Inborn Errors of Metabolism, observed in 56.3% (27 out of 48) of cases. In contrast, 41.7% of patients were born to non-familial unions, and 2% had missing consanguinity data. Subgroup analysis revealed notable differences across metabolic categories. In Organic Acid Disorders (OADs, with 22 patients), 54.5% of patients were born to consanguineous couples. First-cousin marriages accounted for 31.8%, and second-cousin marriages for 22.7%. Additionally, 40.9% of patients were born to non-familial unions. For Amino Acid Disorders (AADs, with 14 patients), consanguinity was present in 50% of cases. First cousins accounted for 28.6%, and second cousins for 21.4%. The remaining 50% of cases were born to non-familial unions. The highest prevalence of consanguinity was observed in Fatty Acid Oxidation Disorders (FAODs, with 12 patients). Two-thirds of cases (66.7%) were born to first-cousin marriages, and the remaining one-third (33.3%) arose from non-familial unions. No second-cousin marriages were reported [Table 3]. These findings suggest that consanguinity may play a notable role in the occurrence of IEMs, with a relatively higher proportion observed among FAOD cases. This highlights the importance of genetic counseling and targeted screening in populations with high rates of familial marriages. The data also suggest that consanguinity may differentially impact the risk of specific metabolic subgroups, providing insights into the genetic factors associated with IEMs.

**Table 3:** Distribution of consanguinity among patients with IEMs

Inborn Errors of Metabolism	First cousin marriges	Second Cousin marriges	Total Consanguinity marriges	None-familial	NA	Total
Organic Acid Disorder	7 (31.8%)	5 (22.7%)	12 (54.5%)	9 (40.9%)	1 (4.6%)	22 (100%)
Amino Acid Disorder	4 (28.6%)	3 (21.4%)	7 (50%)	7 (50%)	0 (0%)	14 (100%)
Fatty Acid Oxidation Disorder	8 (66.7%)	0 (0%)	8 (66.7%)	4 (33.3%)	0 (0%)	12 (100%)
Total	19 (39.6%)	8 (16.7%)	27 (56.3%)	20 (41.7%)	1 (2%)	48 (100%)

Data are presented as number and percentage of cases. Consanguinity marriages include first-cousin and second-cousin marriages in Organic acid disorders, Amino acid disorders, and Fatty acid oxidation disorders

### Geographic distribution of inborn errors of metabolism

**Fig. 6:**
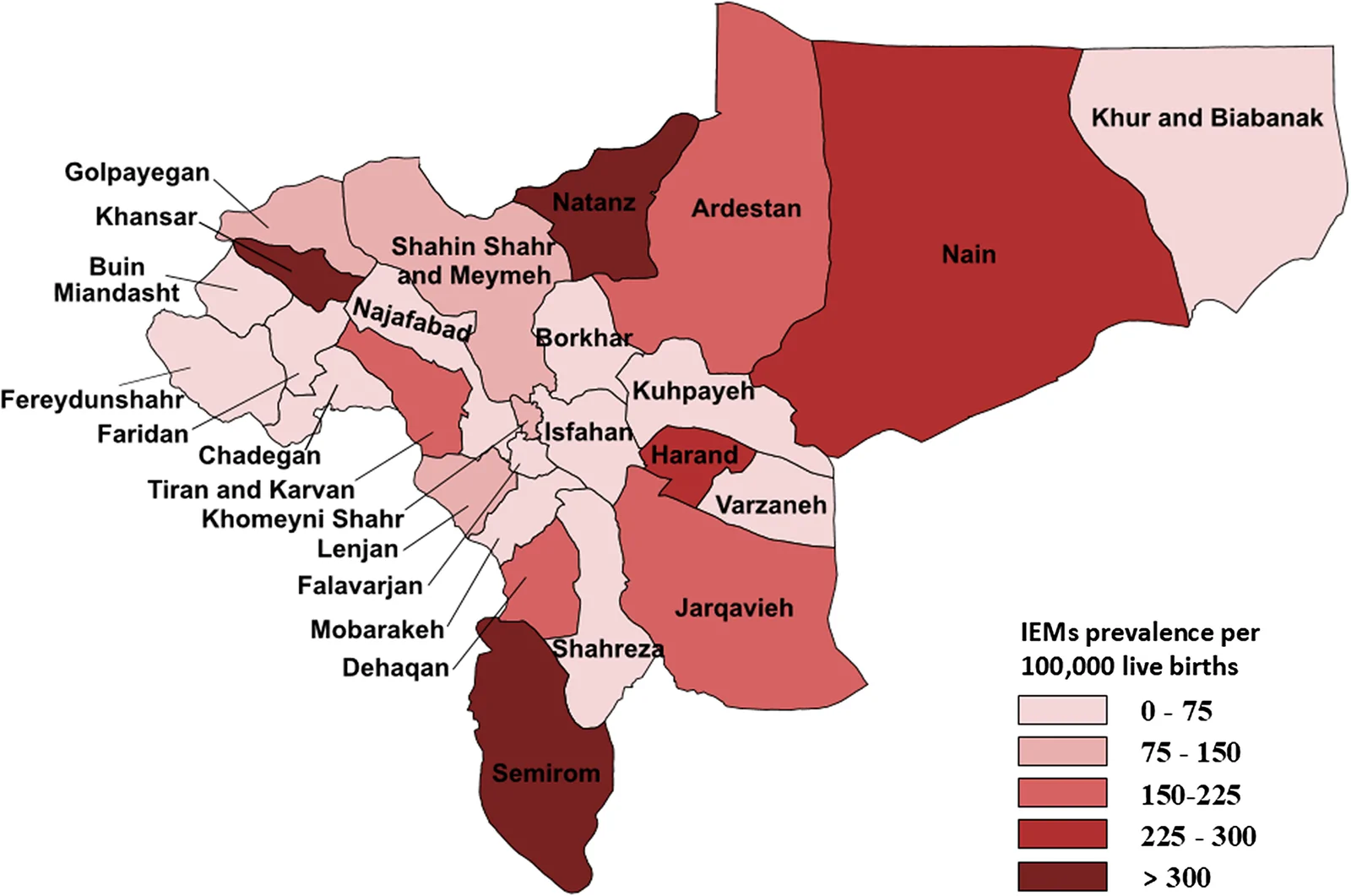
Geographic distribution of inborn errors of metabolism (IEMs) in cities covered by Isfahan University of Medical Sciences. Data are presented as the prevalence per 100,000 live births. Higher prevalence rates were observed in smaller towns and rural areas, whereas major urban centers showed lower prevalence rates. The Small sample size in some counties may have affected the results

**Fig. 7:**
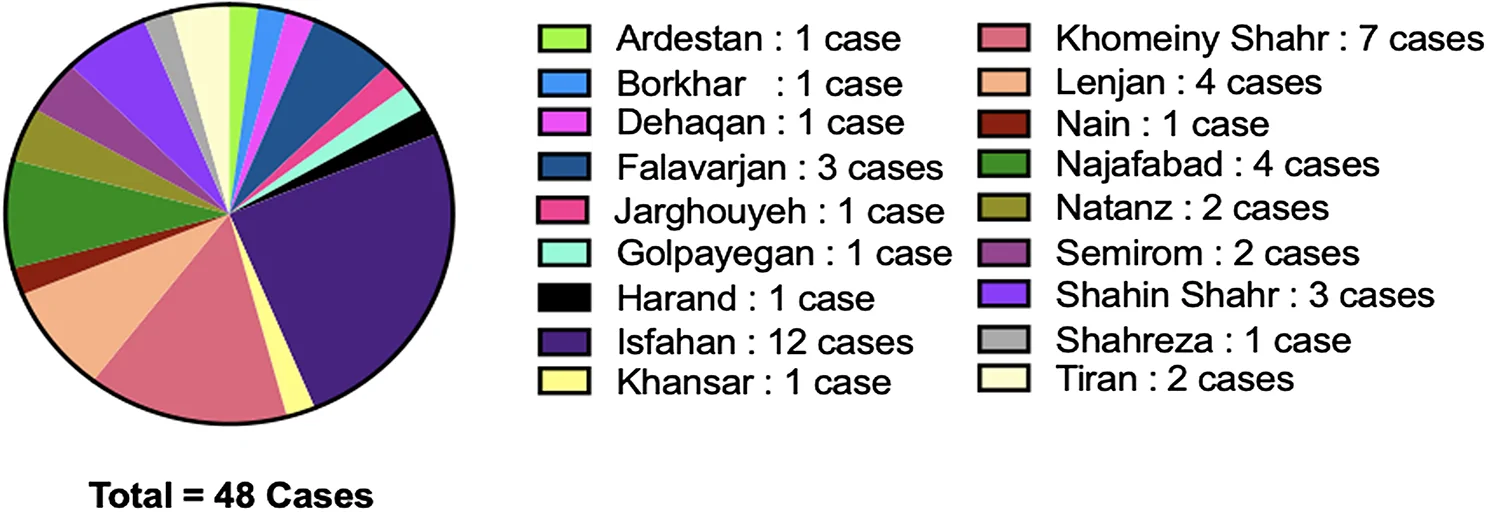
Number of detected IEMs in cities covered by Isfahan University of Medical Sciences. The figure depicts IEM case counts across cities, with the highest numbers in Isfahan (*n* = 12) and “Khomeyni Shahr” (*n* = 7). Cases are also reported in smaller towns, highlighting geographic heterogeneity and the need for targeted screening and genetic counseling programs

The geographic distribution of Inborn Errors of Metabolism across cities covered by Isfahan University of Medical Sciences revealed remarkable heterogeneity in prevalence rates (Figs. 6 and 7; Table 4). The highest prevalence per 100,000 live births was observed in Khansar (384.6), Semirom (339.6), Natanz (322.1), Harand (289.0), and Nain (233.1), indicating potential regional clustering of metabolic disorders. In contrast, major urban centers like Isfahan (38.9) and Najafabad (68.4) showed relatively low prevalence rates despite having a higher absolute number of affected individuals. This suggests a dilution effect due to larger population sizes and increased genetic diversity. Intermediate prevalence rates were observed in Dehaqan (202.8), Jarghouyeh (199.2), Tiran (174.1), Ardestan (167.8), Khomeiny Shahr (117.8), and Lenjan (94.0). Other locations, including Falavarjan (71.1), Borkhar (39.4), Golpayegan (83.5), Shahin Shahr (90.8), and Shahreza (40.3), displayed lower-to-moderate prevalence rates. These patterns collectively suggest that smaller towns and rural areas have higher prevalence rates per capita, possibly influenced by factors like consanguinity, regional founder effects, and limited access to genetic counseling and early screening programs. However, these estimates should be interpreted cautiously because the number of cases in several counties was small, resulting in wide confidence intervals. Although small sample sizes in some counties may have influenced the results, this geographic analysis identifies distinct high-risk areas that may benefit from targeted population screening and preventive genetic interventions, especially in smaller communities with disproportionately high prevalence rates. This data provides critical insights for public health planning and resource allocation to mitigate the burden of IEMs in these regions.

**Table 4:** Birth prevalence of confirmed IEMs by counties affiliated with Isfahan University of Medical Sciences, during 2023–2025, with 95% confidence intervals

County	prevalence rate	95% CI Lower	95% CI Upper	County	prevalence rate	95% CI Lower	95% CI Upper
**Ardestan**	167.8	4.2	934.8	**Khomeini Shahr**	117.8	47.4	242.7
**Borkhar**	39.4	1	219.4	**Khur & Biabanak**	0	0	1128.1
**Buin Miandasht**	0	0	2120	**Kouhpayeh**	0	0	1499.5
**Chadegan**	0	0	796.7	**Lenjan**	94	25.6	240.7
**Dehaqan**	202.8	5.1	1130.2	**Mobarakeh**	0	0	161.9
**Falavarjan**	71.1	14.7	207.7	**Nain**	233.1	5.9	1298.8
**Fereydan**	0	0	414.5	**Najafabad**	68.4	18.6	175.1
**Freydunshahr**	0	0	717.7	**Natanz**	322.1	39	1163.4
**Golpayegan**	83.5	2.1	465.5	**Semirom**	339.6	41.1	1226.6
**Harand**	289	7.3	1610.3	**Shahin shahr**	90.8	18.7	265.3
**Isfahan**	38.9	20.1	67.9	**Shahreza**	40.3	1	224.4
**Jarghouyeh**	199.2	5	1109.9	**Tiran**	174.1	21.1	628.8
**Khansar**	384.6	9.7	2142.9	**Varzaneh**	0	0	809

## Discussion

### Overall prevalence of IEMs

In the present study, the overall birth prevalence of all-cause inborn errors of metabolism (IEMs) in Isfahan Province, Iran, was 67.6 per 100,000 live births (95% confidence interval (CI(: 50.30–89.40), higher than the current global pooled estimate of 50.9 per 100,000 live births (95% CI: 43.4–58.4), although the confidence intervals overlap [1]. This prevalence places Isfahan among areas with relatively higher global rates and agrees with findings reported from other countries in the Eastern Mediterranean region, where the combined prevalence is 75.7 per 100,000 live births (95% CI: 50.0–101.4) [1]. On the other hand, European populations reveal lower prevalence rates, such as approximately 37.4 per 100,000 in Madrid, Spain [14], and approximately 63.4 per 100,000 in large neonatal cohorts in Europe [17]. In addition, a study conducted in eastern coastal areas of China showed that the overall prevalence of IEMs is approximately 38.7 per 100,000 live births [7]. These international differences may be influenced by variations in consanguinity rates, genetic diversity, and newborn screening strategies, including screening panels, diagnostic cut-off values, and case ascertainment protocols.

Within Iran, regional variation is also evident. Compared with the recent newborn screening study from Fars Province [8], which reported a higher overall prevalence of IEMs (100 per 100,000 live births), our study demonstrated a lower prevalence (67.6 per 100,000 live births). Moreover, organic acid disorders were the predominant IEM group in our cohort (45.8%), whereas amino acid disorders predominated in the Fars cohort (39.6%). These differences likely reflect regional variation in population genetic background, founder effects, consanguinity patterns, ethnic composition, and differences in healthcare-related factors, including screening practices. Accordingly, these findings highlight the importance of interpreting regional epidemiological data within their local context rather than extrapolating results from a single province to the entire Iranian population.

### False discovery rate

The high False Discovery Rate (90.8%) is an inherent and expected characteristic of neonatal metabolic screening and has also been reported in newborn screening programs in other countries [7, 12, 13]. These assays are intentionally calibrated for exceptionally high sensitivity to ensure that infants with severe metabolic disorders are not overlooked, which inevitably diminishes specificity. False discovery results can originate from a combination of biological and technical factors, including normal postnatal metabolic fluctuations, prematurity, low birth weight, an immature metabolic system, improper timing of sample collection, contamination, or inadequate drying of blood spot samples, and certain maternal or infant medications or diets. These conditions can transiently alter metabolite levels, mimic disease, and produce abnormal results in otherwise healthy infants.

### Maternal age & IEMs

The highest proportion of IEM cases was observed among mothers aged 30–34 years, followed by those aged 25–29 years, this pattern likely reflects the national distribution of childbearing in Iran, where most live births occur between 25 and 34 years of age [18], rather than an independent effect of maternal age. This interpretation is consistent with the genetic basis of IEMs, as the majority are inherited as autosomal recessive disorders. Consequently, disease occurrence is expected to be driven predominantly by population-specific carrier frequencies and parental consanguinity, both of which are likely to exert a substantially greater influence than maternal age.

### Amino acid disorders (AADs)

An important cause of neonatal morbidity, especially in populations with high rates of consanguineous marriages, is Amino acid disorders. Three important disorders in this group are hyperphenylalaninemia (HPA), classic phenylketonuria (PKU), and tyrosinemia type I (HT1), which are predominantly autosomal recessive genetic diseases [19]. In this study, 14 affected neonates were identified, resulting in an overall prevalence of 19.7 per 100,000 live births (95% CI: 10.78–33.08). This rate exceeds the global birth prevalence of 14.7 per 100,000 live births (95% CI: 12.2–17.2) [1]. The affected newborns were mainly in the normal birth weight range (92.9% between 2,500 and 4,000 g). Most affected neonates were born to mothers aged 18–34 years (78.6%), which aligns with the typical reproductive age in the region. Notably, half of the affected neonates were born to parents with consanguineous marriages, including 28.6% first cousins and 21.4% second cousins. This highlights the probable contribution of autosomal recessive inheritance, compounded by high consanguinity, to the regional burden of amino acid disorders [20]. These findings emphasize the importance of population-specific newborn screening since relying only on clinical signs or demographic risk factors may miss a significant number of affected neonates [21].

Benign hyperphenylalaninemia (HPA), an autosomal recessive disorder, was the most frequent condition. It accounted for 50% of cases (*n* = 7 of 14) with a prevalence of 9.9 per 100,000 live births (95% CI: 3.96–20.31). Global studies report variable prevalences of hyperphenylalaninemia (HPA), ranging approximately from 3.3 to 33 per 100,000 live births in Europe, 10 per 100,000 in France, 5 per 100,000 in the UAE, and 9 per 100,000 in China. These facts highlight regional differences in the prevalence of this disorder [22]. Clinically, HPA is often asymptomatic, though untreated cases with elevated phenylalanine levels may show mild neurocognitive impairment. Plasma phenylalanine thresholds typically guide management [19]. In high-income countries, treatment is typically initiated only when it is clinically justified in order to minimize unnecessary dietary restrictions. In the Middle East countries such as Iran, Turkey, Saudi Arabia, and the UAE, screening programs exist but vary in coverage, follow-up intensity, and intervention thresholds [19]. The predominance of benign HPA in our cohort may reflect the genetic characteristics of the regional population, together with the sensitivity of the screening algorithm for detecting mild elevations of phenylalanine. Similar patterns have been reported in several Middle Eastern populations. However, differences in screening cutoffs and confirmatory protocols across countries should also be considered when comparing prevalence estimates.

Classic phenylketonuria (PKU) accounted for 28.6% of cases, with an prevalence of 5.6 per 100,000 live births (95% CI: 1.53–14.42), while the global prevalence is estimated to be 6.55 per 100,000 live births (95% CI: 5.43–7.76) [1]. In contrast, the Middle East region exhibits some of the highest prevalence rates of PKU, with Egypt, Iran, and Jordan reporting approximately 20 cases per 100,000 live births [23]. Thus, the lower prevalence observed in the current study compared with previous reports may be attributed to improved detection accuracy or limitations related to the short follow-up period. Therefore, further large-scale and long-term studies are warranted to substantiate these findings. Untreated phenylketonuria (PKU) leads to intellectual disability, developmental delay, seizures, eczema, and a distinctive odor. The standard approach for managing PKU worldwide involves a lifelong diet that restricts phenylalanine intake while tyrosine is supplemented, and in some cases, Sapropterin (BH₄) therapy is administered [23]. This strategy has enhanced long-term neurodevelopmental outcomes in countries with well-established newborn screening programs. In the Middle East, national screening has been implemented in numerous countries, but differences in factors such as program consistency, dietary support, and follow-up monitoring impact outcomes [23, 24].

Tyrosinemia type I (HT1) represented 14.3% of cases, with a prevalence of 2.8 per 100,000 live births (95% CI: 0.34–10.18). This rate of prevalence is higher than the global prevalence, which ranges from 0.5 per 100,000 to 1 per 100,000 [25]. Some of the clinical manifestations involve failure to thrive, hepatomegaly, jaundice, coagulopathy, renal tubular dysfunction, and an increased risk of hepatocellular carcinoma if untreated. To improve survival and prevent progression to organ failure, Early initiation of NTBC therapy in conjunction with dietary restriction is needed [26, 27]. Tyrosinemia is included in expanded newborn screening panels in several European countries, the US, and Canada, to reach optimal outcomes [27]. In the Middle East, Tyrosinemia screening has been incorporated into some national programs; however, coverage is inconsistent, and delayed diagnosis continues to be a major challenge [28, 29].

### Organic acid disorders (OADs)

The current study identified 22 patients (45.8%) with Organic Acid Disorders, representing the largest subgroup among all IEMs with an overall prevalence of 31 per 100,000 live births (95% CI: 19.42–46.91). Among these disorders, 2-Methylbutyryl-CoA dehydrogenase deficiency and 3-Methylcrotonyl-CoA carboxylase (3MCC) deficiency were the most prevalent types, each with a prevalence of 7.0 per 100,000 (95% CI: 2.29–16.43), followed by Isovaleric acidemia with a prevalence of 5.6 per 100,000 (95% CI: 1.53–14.42). The overall prevalence of this group of disorders was approximately three times higher than that reported in China (9.9 per 100,000; 95% CI: 7.9–12.6), while the ranking of the most frequent diseases remained remarkably comparable [30]. This difference may reflect regional genetic characteristics, although differences in screening algorithms and confirmatory protocols should also be considered. However, this distribution pattern indicates that OADs constitute a major burden in our region, which aligns with findings reported in Fars province, Iran [8].

Among OADs, 2-Methylbutyryl-CoA dehydrogenase deficiency disrupts the catabolism of isoleucine. It is generally associated with a benign clinical course, although some affected individuals may still be vulnerable to metabolic stress or develop mild symptoms [31]. Moreover, 3-Methylcrotonyl-CoA carboxylase deficiency disrupts leucine degradation. It might predispose affected infants to metabolic crises involving hypoglycemia, ketosis, hypotonia, seizures, and different degrees of developmental delay, which underscores the importance of early recognition and supportive management [32].

The mortality rate within the OAD group was 22.7% (95% CI: 7.4% – 47.7%), representing all recorded deaths in the entire study. The relatively high mortality rate, particularly observed in 3-Methylcrotonyl-CoA carboxylase (3MCC) deficiency, contrasts with several previous studies that question the necessity of newborn screening for 3MCC, and underscores the critical importance of early detection and timely management in improving clinical outcomes [33, 34]. The concentration of all fatalities within the OAD subgroup may suggest that these disorders account for a disproportionate share of severe clinical outcomes and may therefore deserve priority in follow-up programs after newborn screening. It also should be noted that early metabolic crises and diagnostic delays may have contributed to the observed mortality.

Additionally, 22.7% (95% CI: 7.4% – 47.7%) of affected newborns were low birth weight, and 4.5% (95% CI: 0.12% – 21.6% (were very low birth weight, which may indicate an epidemiological correlation with restricted fetal growth [35]. Interestingly, this pattern was observed more frequently among infants diagnosed with Isovaleric Acidemia. However, causality cannot be inferred from these observational data. The relationship between low birth weight and metabolic disorders is not yet clear, and larger studies are needed to better understand this association.

Collectively, these findings note that Organic Acid Disorders remain the most frequent and clinically severe subgroup of IEMs in Isfahan province, characterized by relatively high mortality. Expansion of early neonatal screening and education for high-risk families could substantially reduce morbidity and mortality in this group.

### Fatty Acid Oxidation Disorders (FAODs)

Fatty Acid Oxidation Disorders accounted for 12 cases (25%), with an overall prevalence of 16.9 per 100,000 live births (95% CI: 8.73–29.52). The great majority (75%) were diagnosed with Short-chain acyl-CoA dehydrogenase (SCAD) deficiency, with a prevalence of 12.7 per 100,000 (95% CI: 5.80–24.06), followed by Medium-chain acyl-CoA dehydrogenase (MCAD) deficiency (2.8 per 100,000; 95% CI: 0.34–10.18). These disorders are among the most commonly detected IEMs through screening. The overall prevalence of FAODs has been estimated 6.51 per 100,000 live births (95% CI: 5.14–7.89) in a recent systematic review, which is approximately one-third of the prevalence observed in our cohort. In that review, MCAD was the most frequently reported subtype (5.78 per 100,000; 95% CI: 4.38–7.18) [1]. In contrast, our study revealed a predominance of SCAD deficiency, highlighting a distribution pattern that differs from many other populations where MCAD is typically the leading FAOD [36].

The reported prevalence rate of SCAD deficiency has been 11.8 cases per 100,000 (95% CI: 5.4–25.9 per 100,000) in Sicily through 2017–2020, which aligns with our results [37]. Although the clinical significance of SCAD deficiency has been debated—given its usual nonspecific manifestations and generally benign course—recent evidence suggests that it may be more prevalent than previously recognized [38, 39]. Long-term clinical follow-up will be essential to determine how many of these screen-positive SCAD cases develop clinically significant disease, thereby helping to distinguish true disease burden from detection of biochemical phenotypes with limited clinical relevance.

Unlike the OAD group, no deaths were observed among patients with FAODs in this study. This may be related to the generally mild clinical presentation of SCAD deficiency and the possible benefit of early detection through newborn screening; however, causal relationships cannot be concluded from descriptive data alone. The relatively high number of SCAD cases and the uncertainty of its clinical importance highlight the need for further studies to better understand genotype–phenotype relationships in this population.

### Consanguinity as a risk factor

**Fig. 8:**
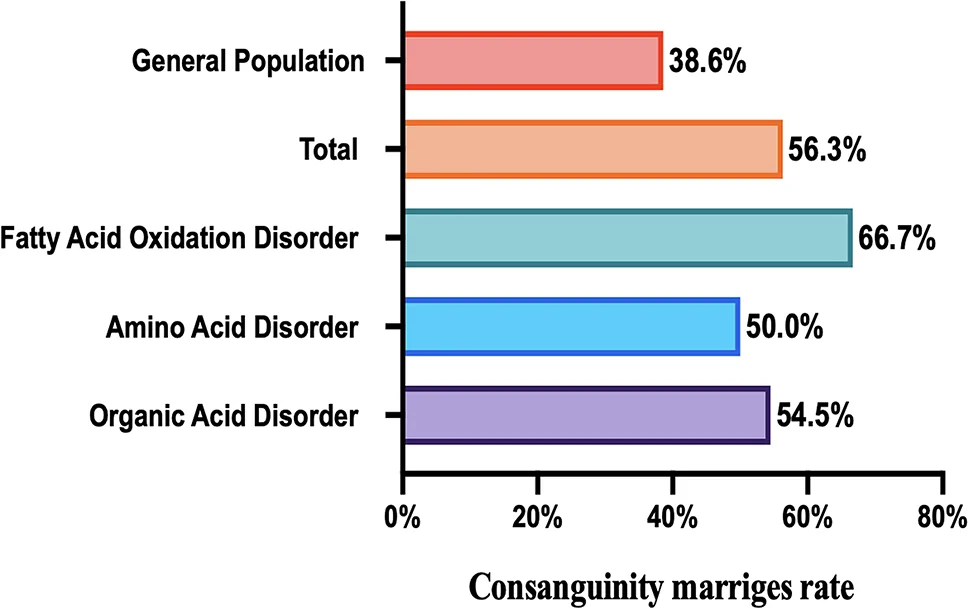
Consanguineous marriage rates among families with IEMs and their subgroups compared with the general population

This study suggests a notable and possible clinically relevant association between parental consanguineous marriages and the prevalence of IEMs (Fig. 8). Consanguineous unions were observed in 56.3% of affected families compared to 38.6% which is Consanguinity marriages rate in Iran as reference population [40], corresponding to a relative risk (RR) of 2.05 (95% [CI]: 1.16–3.62; *p* ≈ 0.017), confirming that consanguinity doubles the population-level risk of IEMs. Fatty acid oxidation disorders carried the highest risk (RR = 3.18, 95% CI: 0.96–10.5; *p* ≈ 0.06), approaching statistical significance, whereas organic acid disorders showed a moderate, non-significant elevation (RR = 1.91, 95% CI: 0.83–4.42; *p* ≈ 0.13), and amino acid disorders exhibited no significant association (RR = 1.59, 95% CI: 0.56–4.53; *p* ≈ 0.38), likely reflecting limited sample size. The elevated prevalence in this region can be attributed to the high frequency of consanguineous marriages, which increases homozygosity for deleterious autosomal recessive alleles and amplifies the population burden of IEM [13, 41, 42]. Importantly, consanguineous marriage, including first- and second-cousin unions, is not unique to Iran and represents a socially and culturally accepted practice in Islamic societies. This pattern is shaped by long-standing cultural traditions, kinship structures, and social preferences that often favor intra-family marriages for economic, social, and familial cohesion reasons.

Accordingly, similar high rates of consanguinity have been reported in multiple populations, including North African countries as well as in Middle Eastern countries including Oman, Saudi Arabia, and the United Arab Emirates, where consanguineous marriages remain relatively common [13, 41, 42]. These findings highlight how consanguinity can influence both individual risk and the overall population burden of IEMs. It underscores the importance of precision screening strategies, genetic counseling, and public health policy in regions with high prevalence.

### Expanded newborn screening

Newborn screening programs constitute one of the most cost-effective preventive strategies in contemporary public health, facilitating the early detection of IEMs before the onset of irreversible clinical manifestations. Early identification through screening not only mitigates morbidity and mortality, but also substantially reduces the long-term economic burden on health systems by preventing delayed diagnosis, recurrent hospitalizations, and lifelong disability care. From a health system perspective, integrating NBS into primary care also signifies a shift towards preventive healthcare, particularly in settings where late diagnosis is common.

In countries where structured screening programs are still in the process of development, including several middle-income settings, the implementation of NBS can be considered a strategic public health investment rather than a purely clinical intervention. Iran, as evidenced by the expanding experience of regional screening initiatives, demonstrates a progressive movement towards strengthening early detection systems, particularly in provinces with established laboratory capacity. Such programs also have significant implications at the family level, as early diagnosis enables timely genetic counseling, informed reproductive decision-making, and reduction of recurrence risk in high-risk families. Overall, expanding NBS coverage in similar settings may improve health outcomes and support long-term healthcare sustainability.

The geographic heterogeneity observed across Isfahan Province suggests that, in addition to consanguinity, local population genetic characteristics may also contribute to IEMs. Historical founder effects and long-standing endogamy in some ethno-religious communities may partly explain the regional clustering of certain disorders through an increased frequency of pathogenic variants. However, these factors were beyond the scope of the present study, as the absence of integrated genomic data precluded precise characterization of pathogenic variants and their population-specific distribution. Moreover, lack of data on access to molecular genetic testing and subsequent clinical follow-up, limited evaluation of the impact of molecular confirmation on patient management and long-term outcomes. In addition, the relatively short study duration limited the assessment of long-term prevalence trends of IEMs. Therefore, extended longitudinal studies incorporating comprehensive molecular and population genetic analyses are warranted to clarify the true disease burden and explain the factors underlying its regional epidemiology.

## Conclusions

The elevated prevalence of inborn errors of metabolism (IEM) in Isfahan is likely attributable to both enhanced local diagnostic capabilities and comprehensive newborn screening programs. These programs facilitate the identification of both prevalent and rare metabolic disorders. These findings underscore the critical need for integrated public health genomics strategies in populations characterized by high consanguinity. Such strategies should encompass expanded newborn and carrier screening, premarital genetic counseling, and population-based next-generation sequencing (NGS) initiatives. The implementation of these measures could significantly enhance early detection, risk stratification, and the prevention of autosomal recessive disorders, ultimately mitigating the burden of IEM at the population level.

## Data Availability

The data that support the findings of this study are available from Deputy of Health, Isfahan University of Medical Sciences; but restrictions apply to the availability of these data, which were used under license for the current study, and so are not publicly available. Data are however available from the corresponding author upon reasonable request and with permission of Deputy of Health, Isfahan.

## Abbreviations

AAD: Amino acid disorder
ACMG: American college of medical genetics and genomics
CI: Confidence interval
DBS: Dried blood spot
FAOD: Fatty acid oxidation disorder
GC/MS: Gas chromatography–mass spectrometry
IEM: Inborn errors of metabolism
LBW: Low birth weight
MS/MS: Tandem mass spectrometry
NBS: Newborn screening
NBW: Normal birth weight
OAD: Organic acid disorder
RR: Relative risk
VLBW: Very low birth weight
